# Vascular Diseases and Gangliosides

**DOI:** 10.3390/ijms20246362

**Published:** 2019-12-17

**Authors:** Norihiko Sasaki, Masashi Toyoda

**Affiliations:** Research team for Geriatric Medicine (Vascular Medicine), Tokyo Metropolitan Institute of Gerontology, Sakaecho 35-2, Itabashi-ku, Tokyo 173-0015, Japan

**Keywords:** vascular disease, atherosclerosis, ganglioside, vascular cells, inflammatory cells, aging, senescence

## Abstract

Vascular diseases, such as myocardial infarction and cerebral infarction, are most commonly caused by atherosclerosis, one of the leading causes of death worldwide. Risk factors for atherosclerosis include lifestyle and aging. It has been reported that lifespan could be extended in mice by targeting senescent cells, which led to the suppression of aging-related diseases, such as vascular diseases. However, the molecular mechanisms underlying the contribution of aging to vascular diseases are still not well understood. Several types of cells, such as vascular (endothelial cell), vascular-associated (smooth muscle cell and fibroblast) and inflammatory cells, are involved in plaque formation, plaque rupture and thrombus formation, which result in atherosclerosis. Gangliosides, a group of glycosphingolipids, are expressed on the surface of vascular, vascular-associated and inflammatory cells, where they play functional roles. Clarifying the role of gangliosides in atherosclerosis and their relationship with aging is fundamental to develop novel prevention and treatment methods for vascular diseases based on targeting gangliosides. In this review, we highlight the involvement and possible contribution of gangliosides to vascular diseases and further discuss their relationship with aging.

## 1. Introduction

**Figure 1:**
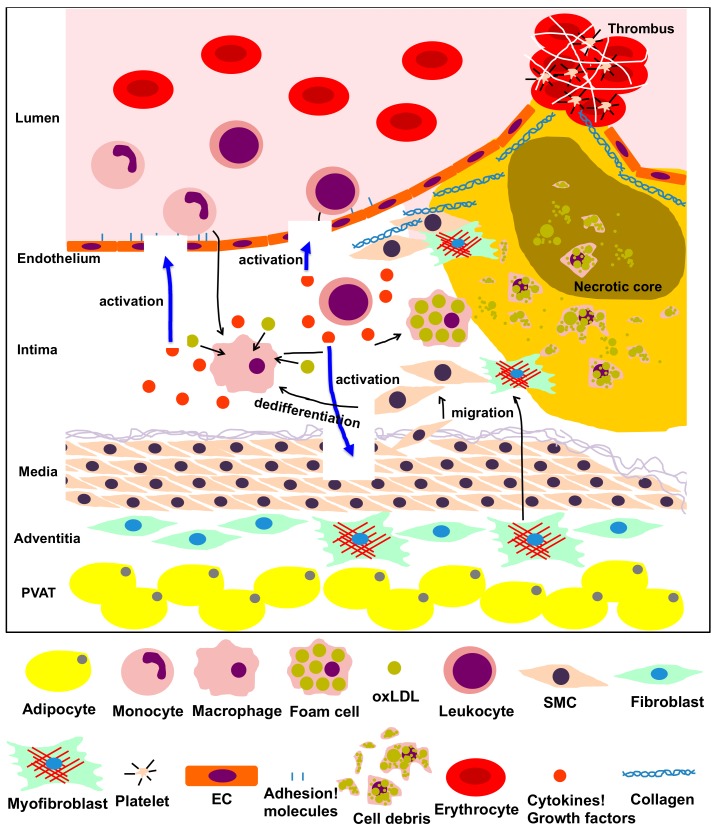
The process of atherosclerosis involving vascular and vascular-associated cells. Vascular cells, including endothelial cells (ECs), smooth muscle cells (SMCs), fibroblasts, adipocytes from the intima, media, adventitia and perivascular adipose tissue (PVAT), and other inflammatory cells participate in the inflammatory process of atherosclerosis via multiple intricate pathways. Dysfunction of ECs, transformation of monocytes/macrophages into foam cells, migration, proliferation and dedifferentiation of smooth muscle cells (SMCs), transformation of fibroblasts into myofibroblasts, and production of adipokines by adipocytes in the PVAT are predominantly implicated in the pathological process of atherosclerosis. This process is characterized by the following steps: atheromatous plaque formation, plaque failure and thrombus formation.

Vascular diseases, including myocardial infarction and cerebral infarction, are among the main causes of death worldwide and are mainly caused by atherosclerosis [1]. Atherosclerosis is the most typical form of arteriosclerosis, a condition that results in thickening and loss of elasticity in the arterial wall. There are various causes for atherosclerosis, many of which are closely related to lifestyle and lifestyle-related diseases. These causes include diabetes, hyperlipidemia, hypertension, smoking, and stress [2]. Since atherosclerosis is particularly increased in elderly people, aging has been considered as an initiating and developmental factor for vascular diseases [2]. However, the molecular mechanisms by which aging promotes vascular diseases are not well understood. Several types of cells, such as endothelial cells (ECs), inflammatory cells, vascular smooth muscle cells (VSMCs) and fibroblasts, are involved in atherosclerosis. Atherosclerosis is characterized by the following steps: (1) atheromatous plaque formation, (2) plaque failure and (3) thrombus formation (Figure 1). Each step is described below.

*(1) Atheromatous plaque formation*. Dysfunction of ECs (caused by factors like obesity and diabetes mellitus) leads to upregulation of adhesion molecules on the cellular membrane, generation of inflammatory cytokines and an increase in vascular permeability of lipoproteins [3]. Migration of monocytes into the intima is often accompanied by their differentiation into macrophages and internalization of atherogenic lipoproteins through upregulated scavenger receptors. After internalizing lipoproteins, most macrophages transform into foam cells. Foam cells aggregate to form the atheromatous core, leading to the formation of atheromatous plaques that include lipids, cholesterol crystals and cell debris [4]. VSMCs can migrate into the intima, proliferate excessively and promote synthesis of extracellular matrix (ECM) and lipid deposition, inducing fibrosis, thickening of the arterial wall and luminal stenosis [5]. Additionally, fibroblasts in the adventitia can differentiate into myofibroblasts, migrate into the intima and contribute to collagen deposition and neointimal expansion [6]. Activated mast cells in the sub-endothelium can also cause plaque progression by exocytosis of granules containing effector molecules, which stimulate leukocyte recruitment and lipid accumulation [7]. CD4^+^ T cells, once activated by oxidized low-density lipoprotein antigens, initiate the formation and propagation of the atheroma by recruitment of macrophages to the plaque and enhanced formation of foam cells [8]. In perivascular adipose tissues, dysfunction of adipocytes leads to the secretion of pro-inflammatory adipokines, resulting in EC dysfunction, infiltration of inflammatory cells and initiation of atherosclerosis [9].

*(2) Plaque failure*. Endothelial to mesenchymal transition (EndMT) is the cause of several cardiovascular diseases [10,11]. Plaque hypoxia promotes EndMT and in turn, EndMT-derived fibroblast-like cells produce high levels of matrix metalloproteinases (MMPs), leading to plaque rupture [12]. Moreover, inflammatory cells (T cells, macrophages, neutrophils, etc.) that accumulate in plaques produce cytokines required for EndMT (such as transforming growth factor [TGF]-β1), causing plaque instability [13]. At the same time, the extracellular matrix produced by VSMCs strengthens the fibrous cap of the atheromatous plaque to protect against plaque rupture and thrombosis [5]. Thinning of the cap is the prelude to the subsequent rupture of the plaque. Matrix-degrading metalloproteinases, which are mainly produced by macrophages and foam cells in the plaque, contribute to cap thinning [14], while mast cells activated by psychological stress destabilize the plaque [7,15]. Finally, activated CD8^+^ T cells cause rupture of the developed atheroma by secretion of the serine protease granzyme B, which induces apoptosis in VSMCs, leading to plaque destabilization in atherosclerotic lesions [16].

*(3) Thrombus formation*. Upon dysfunction of ECs, the physiological balance between antithrombotic and thrombotic molecules is disrupted, resulting in increased levels of thrombotic substances, such as the von Willebrand factor, and attenuation of antithrombotic substances, such as heparin. These processes facilitate thrombosis with devastating consequences [17]. At the site of plaque rupture, platelets bind to ECM components like fibrillar collagen, accumulate and exocyte granules upon activation, contributing to the thrombotic process [18]. Circulating inflammatory cells like neutrophils also play a crucial role in atherothrombosis. Activated neutrophils interact with thrombin-activated platelets and release chromatin, nuclear proteins and serine proteases extracellularly, promoting thrombus formation [19].

**Figure 2:**
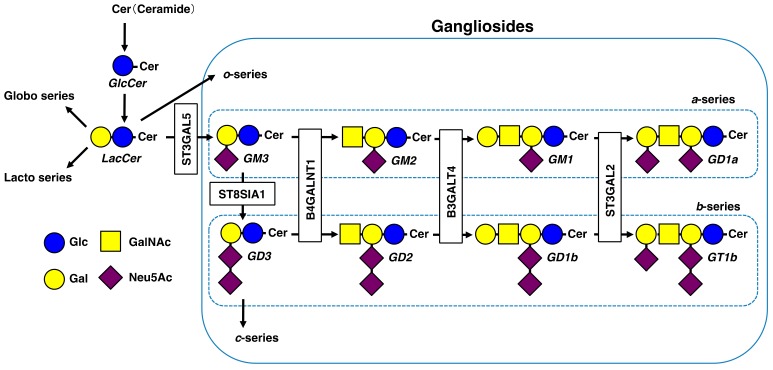
Schematic diagram of GSL pathways. Pathways of the major gangliosides (*a*- and *b*-series) mentioned in this review are shown within the dotted rectangles. Glc, Glucose; Gal, Galactose; GalNAc, *N*-acetylgalactosamine; Neu5Ac, *N*-acetylneuraminic acid.

To develop effective prevention and treatment methods for vascular diseases, specific markers need to be identified. Gangliosides, a group of glycosphingolipids (GSLs), are cell surface sugar chain markers (Figure 2). Gangliosides are acidic GSLs with one or more sialic acid residues on their carbohydrate moieties. They are mainly located in lipid rafts, which are also enriched with cholesterol, phospholipids and raft-associated proteins [20]. Lipid rafts play important roles in cell signaling pathways and physio-pathological conditions. Under physio-pathological conditions, changes in ganglioside levels affect the localization of raft-associated cell surface proteins and lead to reduction of membrane fluidity, causing cellular dysfunctions [21,22,23]. According to Svennerholm [24], gangliosides are classified in four series (*o*-, *a*-, *b*- and *c*-series). The *a*-series gangliosides GM1, GM2, GM3 and GD1a and *b*-series gangliosides GD3, GD2, GD1b and GT1b have been characterized in several types of tissues and cells, such as vascular and inflammatory cells [25].

In this review, we highlight the involvement and possible contribution of gangliosides to vascular diseases and further discuss their relationship with aging.

## 2. Gangliosides in Vascular and Vascular Associated-Cells (Table.1)

As described above, blood vessel-constituting cells (ECs, VSMCs, fibroblasts) can be involved in atherosclerosis, leading to vascular diseases. Gangliosides are expressed on these cells (see Table 1) and their relevance to vascular diseases is detailed below.

### 2.1. ECs and Gangliosides

To date, several gangliosides have been reported to be expressed on ECs. In bovine aortic endothelial cells (BAECs), GM3 and GM1 are endogenously expressed [57]. Endogenous cell surface GM1 functions as a coreceptor for basic fibroblast growth factor (bFGF) in transformed fetal bovine aortic endothelial GM 7373 cells [26]. Exogenous addition of GM1 or GM2 inhibits bFGF-induced proliferation of BAECs, whereas GM3 enhances bFGF-induced proliferation [27]. In human umbilical vein endothelial cells (HUVECs), exogenous addition of GD1a enhances vascular endothelial growth factor (VEGF)-induced signaling, involved in proliferation and migration [28]. In contrast, exogenous addition of GM3 inhibits angiogenesis via inhibition of the binding of VEGF to VEGF receptor (VEGFR)-2 and induction of VEGFR dimerization [29]. In addition, Kim et al. reported that exogenous addition of GM3 inhibits VEGF-induced intracellular adhesion molecule-1 (ICAM-1) and vascular cell adhesion molecule-1 expression, leading to reduced monocyte adhesion to HUVECs [30]. Furthermore, they showed that pre-injection of GM3 in mice inhibits VEGF- and VEGF/tumor necrosis factor alpha (TNFα)-induced expression of adhesion molecules in vein tissues [30]. In human aortic endothelial cells (HAECs), *a*-series gangliosides GM1 and GD1a and *b*-series gangliosides are expressed on the cell surface [31]. To identify the specific gangliosides contributing to EC dysfunction in aging, we investigated the effects of changes in individual cell surface gangliosides in HAECs. We found that GM1 expression increases with cellular senescence on the cell surface of HAECs. Increased GM1 levels do not affect the induction of cellular senescence. On the other hand, they lead to a decrease in insulin signaling related to reduced nitric oxide (NO) production. In addition, GM1 expression is high in HAECs derived from elderly people, suggesting its involvement not only in cellular senescence, but also in the decrease in endothelial function that accompanies aging. These results show that GM1 is involved in endothelial function during cellular senescence and aging and is closely linked to vascular disease [23,31].

Vascular insulin resistance induced by inflammatory cytokines is associated with the initiation and development of vascular diseases. In humans, circulating TNFα levels increase with aging [58], suggesting a correlation between vascular insulin resistance and plasma TNFα levels. We showed in HAECs stimulated with TNFα that GM1 expression levels on cell membranes change depending on time of exposure and concentration of TNFα and are associated with the regulation of the insulin signaling cascade [32]. These results suggest that cell surface GM1 is a key player in the induction of vascular insulin resistance mediated by TNFα during inflammation. Thus, GM1 has great potential as an EC extracellular target for prevention and cure of vascular diseases [23].

Numerous studies have demonstrated that ECs are capable of undergoing EndMT, a newly recognized type of cellular trans-differentiation [10,11]. EndMT-derived cells have typical mesenchymal morphology and functions, such as acquisition of movement ability and contractile properties. EndMT is considered to participate in the pathogenesis of several cardiovascular diseases. However, to date, no report is available on the involvement of gangliosides in EndMT. Epithelial cells can undergo a process called epithelial-mesenchymal transition (EMT), which is similar to EndMT. In the human epithelial lens cell line HLE-B3, TGF-β1, one of the EMT up-regulators induces higher expression of GM3. In turn, interaction of GM3 with the TGF-β−receptor promotes EMT [59]. Among breast cancer stem cells, a small portion of cells exhibits co-expression of ganglioside GD2 and CD44high/CD24low. GD2 expression is associated with EMT induction [60]. Based on these findings, the contribution of gangliosides to EndMT could be at least speculated. Future studies are needed to clarify the relationship between gangliosides and EndMT and thus, aid the development of novel prevention and therapeutic strategies for vascular diseases.

### 2.2. VSMCs and Gangliosides

VSMC proliferation is associated with the development and progression of cardiovascular diseases. GD3 has a dual role in modulating proliferation and apoptosis of VSMCs [33], and increased levels of GD3 are known to be associated with atherosclerosis [61]. Overexpression of GD3 attenuates platelet-derived growth factor (PDGF)-induced activation of the extracellular signal-regulated kinase (ERK) pathway and suppresses the proliferation of mouse VSMCs [34]. Furthermore, overexpression of GD3 leads to inhibition of TNFα-induced MMP-9, which is implicated in the progression of atherosclerotic lesions [62]. In addition, several studies have shown the accumulation of GM3 in atherosclerotic lesions [63]. A study carried in mouse VSMCs has shown that TNFα-induced proliferation and induction of MMP-9 are inhibited upon GM3 overexpression. In this study, treatment with anti-GM3 antibodies reversed the inhibitory effects of GM3, indicating that GM3 controls VSMC proliferation and migration during the formation of atherosclerotic lesions [64]. In contrast, in rat aortic VSMCs, exogenous addition of GM1 and GM2, but not GM3, induces activation of the ERK pathway and promotes VSMC proliferation [35].

VSMCs are not terminally differentiated and can change their phenotype in response to environmental cues, such as growth factors/inhibitors, mechanical influences, cell–cell and cell–matrix interactions, extracellular lipids and lipoproteins, and various inflammatory mediators present in the injured artery wall [65]. Dedifferentiation of VSMCs into macrophage-like cells can be promoted by activation of Krüppel-like factor 4, which is one of the pluripotency transcription factors controlled by PDGF-BB signaling [66,67]. In the neuroblastoma cell line SH-SY5Y, exogenous addition of GM1, GM2, GD1a or GT1b inhibits phosphorylation of the PDGF receptor (PDGFR), resulting in suppressed cell growth, whereas growth inhibition mediated by exogenous GM3 acts downstream of PDGF signal transduction [68]. In Swiss 3T3 cells, overexpression of GM1 by transfection of β4GalNAcT1 and β3GalT4 inhibits PDGF-BB-stimulated growth due to PDGFR dispersion from lipid rafts [69]. Furthermore, exogenous addition of GM1 promotes the osteogenic differentiation of human tendon stem cells via reduction of PDGFR phosphorylation [70]. On the other hand, the contribution of gangliosides to PDGF-BB signaling in VSMCs still has to be clarified, but it could be speculated from the reports cited above that gangliosides are involved in PDGF-BB signaling-mediated dedifferentiation of VSMCs.

### 2.3. Fibroblasts and Gangliosides

In human fibroblasts derived from fetal lung, GM3 and GD3 are the most commonly expressed gangliosides and their expression decreases in long-term cultures, in which cells undergo senescence [71]. Exogenous GM3 and GD1a promote epithelial growth factor (EGF)- or bFGF-stimulated proliferation of normal human dermal fibroblasts [36]. Additionally, GM3 synthase-deficient skin-derived human fibroblasts exhibit reduction of EGF-stimulated proliferation and migration [37]. In contrast, embryonic fibroblasts derived from GM3 synthase knock-out mice exhibited higher growth potential than wild-type cells due to suppression of the MAPK pathway [39]. GD3 is a structural component of the autophagosome and exogenous administration of GD3 activates autophagy in normal human skin-derived fibroblasts [38]. In rat heart fibroblasts, exogenous GM1 protects from apoptosis through induction of the synthesis of sphingosine 1-phosphate [40]. Despite the functional role of gangliosides in fibroblasts has been demonstrated, the specific type of gangliosides and organs from which fibroblasts originate need to be taken into account. In fact, different organ-derived fibroblasts, such as skin- and oral-derived fibroblasts, exhibit different levels of hyaluronic acid and different growth responses upon TGF-β1 stimulation [72]. Therefore, further functional investigation of gangliosides (particularly in heart-derived fibroblasts) is required to clarify their contribution to cardiovascular diseases.

During atheromatous plaque formation, myofibroblasts differentiated from fibroblasts elicit collagen deposition and neointimal expansion in the intima. TGF-β1 signaling is known to regulate myofibroblast differentiation [73]. As described above, GM3 and GD2 contribute to TGF-β1 signaling [59,60]. In addition, raft GM1 is important for TGF-β1-stimulated myofibroblast differentiation in human skin-derived fibroblasts [74]. To date, the direct contribution of gangliosides to myofibroblast differentiation has not been clarified. Therefore, further studies are required, although it can be speculated that gangliosides GM3, GM1 and GD2 are involved in myofibroblast differentiation.

### 2.4. Inflammatory Cells and Gangliosides

In human neutrophils, expression of GSLs is heterogeneous and complex ganglioside mixtures, including GM1 and GM3, exist [41]. Mature neutrophils express the highest levels of GM1 [41,42]. Furthermore, when cells undergo apoptosis, expression of GM1 at the cell surface is lost at an early stage. Thus, GM1 is considered a marker for detection of aged neutrophils [43].

It is well known that GD3 is the most abundantly expressed ganglioside on the surface of almost all mast cells [75]. It has also been shown that elevated expression levels of GM3, GM2, GM1 and GD1a can be observed during maturation of the human mast cell line HMC-1 [44] and that exogenous GM3 inhibits interleukin (IL)-3-stimulated cell proliferation of bone marrow-derived mouse mast cells [45]. In addition, it has been reported that cross-linking of GD1b-derived gangliosides activates RBL-2H3, a rat mast cell line, leading to the release of inflammatory cytokines, such as IL-4, IL-6 and TNFα [46].

Monocytes and macrophages express high levels of GM3 in both humans and mice [76]. Cultured human macrophages yield about seven times the amount of GM3 (per million cells) of peripheral blood monocytes [77]. In the human pre-myeloid leukemia cell line HL-60 and histiocytic lymphoma cell line U937, exogenous GM3 induces monocytic cell differentiation and notably, GM3 increase during macrophage-like cell differentiation [47]. GM3 synthase levels are significantly higher in human monocyte-derived macrophages than in monocytes and GM3 has been considered as a physiological modulator of macrophage differentiation in human atherosclerotic aorta [78]. In bone marrow-derived macrophages, peritoneal macrophages and the Raw264.7 macrophage cell line, exogenous GM1 contributes to the induction of arginase-1, a major M2 macrophage marker, and to the secretion of monocyte chemoattractant protein-1 (MCP-1) through CD206-mediated activation of signal transducer and activator of transcription (STAT) 6 [48].

Human T cells express both GM3 and GM1, which are clustered in lipid rafts and considered to be involved in T cell activation [49]. Furthermore, other gangliosides (GD1a, GD1b, GT1b, etc.) have been detected at minor levels in human T cells [79,80]. It has been demonstrated that GM1 expression is upregulated in human CD8^+^ T cells upon IL-2 stimulation [50]. In human CD4^+^ T cells, exogenous GM3 and GM1 downregulate the cell surface expression of CD4, inhibiting lymphocyte function-associated antigen-1-dependent adhesion [51]. In murine T cells, GM3, GM1, GD1b and GD3 are expressed similarly to human T cells. Murine CD4^+^ T cells express higher levels of ST3GAL5 than CD8^+^ T cells to synthesize *a*- and *b*-series gangliosides (GM1 and GD1b). In contrast, murine CD8^+^ T cells express more B4galnt1, resulting in higher levels of *o*-series gangliosides [81].

Taken together, these data show that gangliosides in inflammatory cells are prominently involved in atherosclerosis.

### 2.5. Other Types of Cells and Gangliosides

In human platelets, GM3 is major ganglioside and GD3 is synthesized after activation [52]. Additionally, exogenous GM3 and GM1 induce the activation of human platelets, resulting in Ca^2+^ mobilization and shape change [54]. GD3 selectively stimulates human platelet adhesion, spreading and aggregation [53]. Kim et al. showed that exogenous GD2 induces apoptosis in human platelets by cross-linking Siglec-7 [55].

In 3T3-L1 mouse adipocytes, increased expression of GM3 upon TNFα stimulation induces insulin resistance through interaction between GM3 and the insulin receptor [56]. Furthermore, GM3 expression is elevated upon inflammatory conditions in primary mouse adipocytes and adipose tissues [82]. Insulin resistance in mouse adipocytes causes the production of MCP-1, which recruits monocytes and activates proinflammatory macrophages [83].

## 3. Relevance to Aging

Aging is one of the main risk factors for the onset and progression of vascular diseases [84]. In the human body, senescent cells accumulate spontaneously with aging. The main characteristics of senescent cells are: permanent arrest of the cell cycle, enlarged and flattened morphology, high production of various physiologically active factors, known as senescence-associated secretory phenotype (SASP) [85]. SASP factors derived from senescent cells cause chronic inflammation and lead to disease. Therefore, it has been assumed that senescent cells are involved in age-related diseases, including vascular diseases. In fact, recent reports have shown the involvement of senescent cells in vascular diseases through the use of senolytic drugs targeting senescent cells and studies targeting the senescence marker p16^Ink4a^ [86,87]. Increased expression of p16^Ink4a^ and senescence-associated β-galactosidase activity are markers for senescent cells, but cell surface markers specific for senescent cells have not been identified yet. In particular, there is no specific marker available for senescent cells associated with vascular diseases. As we have already suggested, gangliosides are good candidate cell surface markers for senescent cells associated with vascular diseases.

**Figure 3:**
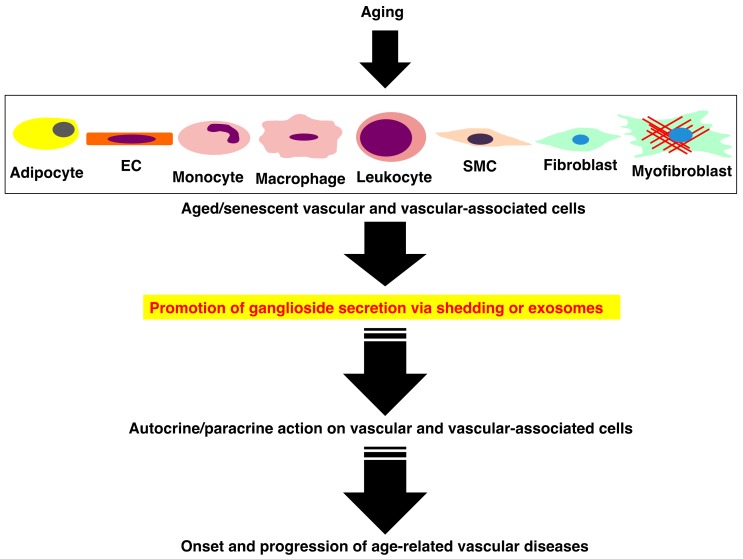
Possible involvement of gangliosides derived from aged and senescent cells in the onset and progression of age-related vascular diseases.

In HAECs, ICAM-1 expression is increased upon cellular senescence and NO activity is reduced compared with young cells [88]. Aged VSMCs exhibit enhanced inducible NO synthase (NOS) activity and higher expression of ICAM-1 [89] and senescent VSMCs release SASP factors including IL-6 [90]. In macrophages, senescence promotes IL-4-induced polarization and attenuation of the JAK2-STAT1 pathway [91]. Thus, these data indicate that senescence of vascular and inflammatory cells is involved in the progression of atherosclerosis, despite the molecular mechanisms of this process, are not well understood. In vascular, inflammatory and other types of cells, senescence is induced with aging and subsequently, these senescent cells may secrete gangliosides via shedding or exosomes. It is known that secretion of exosomes containing gangliosides is increased in senescent cells [92] and that gangliosides can be incorporated into other cells via exosomes [93]. Therefore, there is the possibility that gangliosides derived from senescent cells may act in an autocrine/paracrine manner on their respective cell groups via shedding or exosomes and thus be involved in the onset and progression of age-related vascular diseases (Figure 3).

In conclusion, we introduced the fact that gangliosides are expressed on vascular and vascular-associated cells and described possible contribution of gangliosides to atherosclerosis leading to vascular diseases. Furthermore, aging will affect the expression of gangliosides on those cells, resulting in the onset and progression of age-related vascular diseases. Expression levels of gangliosides change with aging and senescence in HAECs [31] and fibroblasts [71]. However, only a few reports have investigated the relationship between aging/senescence and gangliosides, and the details of this relationship are far from being fully understood. Future studies confirming that gangliosides are directly implicated in aging and senescence may finally identify gangliosides as novel targets for prevention and treatment of age-related and vascular diseases.

## Figures and Tables

**Table 1:** Functional roles of endogenous or exogenous gangliosides in vascular and vascular-associated cells.

Cell Type	Sources	Types of Gangliosides	Functional Roles	References
GM 7373 cells (ECs)	Bovine	GM1	Coreceptor of bFGF	[26]
BAECs	Bovine	GM2, GM1	Inhibition of proliferation	[27]
		GM3	Promotion of proliferation	[27]
HUVECs	Human	GD1a	Enhancement of VEGF-induced signaling, proliferation and migration	[28]
		GM3	Inhibition of VEGF signaling, angiogenesis and adhesion molecules	[29,30]
HAECs	Human	GM1	Association with aging and Inhibition of insulin signaling	[31,32]
VSMCs	Human	GD3	Modulation of proliferation and apoptosis	[33]
VSMCs	Mouse	GD3	Inhibition of PDGF-induced ERK pathway and proliferation	[34]
		GD3	Inhibition of TNFα-induced MMP9 expression	[34]
VSMCs	Rat	GM2, GM1	Activation of ERK pathway and promotion of proliferation	[35]
Fibroblasts (dermal)	Human	GM3, GD1a	Promotion of EGF or bFGF stimulated proliferation	[36,37]
		GD3	Activation of autophagic process	[38]
Fibroblasts (embryonic)	Mouse	GM3	Attenuation of FBS stimulated MAPK pathway	[39]
Fibroblasts (heart)	Rat	GM1	Protection from apoptosis caused from protein kinase C inhibition	[40]
Neutrophils	Human	GM1	Association with maturation	[41,42]
		GM1	Decrease at early stage of apoptosis	[43]
HMC-1 (mast cell line)	Human	GM3, GM2, GM1, GD1a	Association with maturation	[44]
Mast cells	Mouse	GM3	Inhibition of IL-3 stimulated proliferation	[45]
RBL-2H3 (mast cell line)	Rat	GD1b	Activation and induction of inflammatory cytokines	[46]
HL-60, U937 (monocyte)	Human	GM3	Induction of cell differentiation	[47]
Raw264.7 (macrophage)	Mouse	GM1	Induction of arginase-1 and MCP-1	[48]
T cells	Human	GM3, GM1	Association with activation	[49]
CD8^+^ T cells	Human	GM1	Increase with IL-2 stimulation	[50]
CD4^+^ T cells	Human	GM3, GM1	Downregulation of CD4 expression	[51]
Platelets	Human	GD3	Association with activation	[52,53]
		GM3, GM1	Induction of activation with Ca^2+^ mobilization and shape change	[54]
		GD2	Induction of apoptosis	[55]
3T3-L1 (adipocyte)	Mouse	GM3	Inhibition of insulin signaling	[56]
